# Prolonged-Release Buprenorphine Therapy in Opioid Use Disorder Can Address Stigma and Improve Patient Quality of Life

**DOI:** 10.7759/cureus.18513

**Published:** 2021-10-05

**Authors:** Lorenzo Somaini, Sarah Vecchio, Camilla Corte, Carmen Coppola, Aisling Mahony, Alexandra Pitts, Manuela Cutuli, Rosetta Orso, Richard Littlewood

**Affiliations:** 1 Addiction Treatment Centre, Local Health Unit, Azienda Sanitaria Locale di Biella, Biella, ITA; 2 Research, Applied Strategic, London, GBR; 3 Consulting, applied strategic, London, GBR

**Keywords:** injected, buprenorphine, innovation, lived experience, opioid agonist therapy, opioid epidemic

## Abstract

Treatment for opioid use disorder (OUD) including opioid agonist therapy (OAT) is effective. Medication with the oral administration of methadone and buprenorphine has well-known limitations (establishing consistent optimal dosing levels, misuse, diversion, and accidental exposure). Treatment may require attendance at treatment services for collection and consumption of medication; this is associated with stigma and discrimination. Novel therapeutic options include approved, injectable, prolonged-release buprenorphine (PRB) products providing consistently optimal drug levels and less frequent dosing. This work assesses the lived experience of persons currently engaged in OUD therapy to define the potential value of novel therapeutic options in order to inform treatment decisions.

One hundred and twenty-two people engaged with treatment services participated in this assessment. Seventy-two percent of participants believed that novel therapeutic options would improve quality of life and 67% stated it would reduce stigma and discrimination. Participants were neither concerned about the efficacy of (net score negative 30%), or lack of control over (net score negative 36%) treatment, nor about reduced contact with treatment services (net score negative 11%). Results from this assessment indicate that the provision of choice including novel therapeutic options is likely to improve quality of life and reduce the stigma of persons with OUD.

## Introduction

Opioid use disorder (OUD) is associated with low social equity, discrimination and disadvantage [1-6], and current treatment strategies - although effective in reducing harm to the individual and society - may amplify these challenges. Conventional treatment programs based on oral administration include daily visits to treatment services for collection of, and/or consumption of medication; this is associated with limitations including stigma and reduced quality of life. Oral therapy may also be associated with therapeutic challenges (achieving consistent drug levels, adherence to treatment regimens) and other issues (exposure to minors, misuse, diverting of administered medication) [7-9].

Established oral therapeutic options used in treatment programs for OUD include methadone and buprenorphine. Novel therapeutic options include prolonged-release buprenorphine products (PRB), which are administered weekly or monthly. PRB can reduce negative outcomes [10-15], is proven to be effective, and is associated with benefits (less discrimination and improved quality of life) [16-18]. It provides consistent levels of the drug through its prolonged-release mechanism, enabling correct dosing throughout treatment [16,17]. This reduces the frequency of attendance to treatment services, which can lead to less discrimination and stigma, and potentially more persons with OUD seeking treatment.

PRB products are available in the USA and Europe [13]. These products show comparable efficacy and safety in the treatment of OUD in various settings and different communities including prisons [19-21]. This work aims to understand, from lived experience, the benefits of injected novel therapeutic options including possible improvements in quality of life and reduction in stigma associated with treatment.

## Materials and methods

An assessment of lived experience by a survey of persons engaged with OUD treatment was developed following an established example [7]. This assessment was based on 25 questions including a description of participants (demographics, substance use history, and current OUD treatment; current treatment experience, assessment of the potential of novel therapeutic options such as PRB). The focus was on the quality of life, clinical efficacy, and stigma. This assessment was conducted in an Italian addiction center. The survey was distributed and implemented in a similar method to a previous example [7]. Sample determination followed the previously defined method [7]. Consent was obtained from all participants prior to beginning the assessment.

Participants reviewed statements describing their lived experience in OUD care and indicated agreement or disagreement using a 7-point Likert scale (disagree strongly, disagree moderately, disagree slightly, neutral, agree slightly, agree moderately, agree strongly). Analysis of results was completed by combining scores for each statement; to obtain an overall net response score of the difference between the percentage of total positive responses and total negative responses for each statement was determined [22]. This net score represents the level of agreement or disagreement with the statement.

## Results

Population

One hundred and twenty-two people currently receiving therapy in treatment programs for OUD participated in the assessment (Table 1). The average duration of opioid use experience was 16 years. A majority of participants were males (73%) and between 25 and 50 years old (80%). All were engaged in treatment services, receiving OAT prescriptions (56% methadone, 43% buprenorphine). The most common goal for engaging in treatment was to stop using all drugs and medication (71%), 24% wanted to stop using illicit drugs but remain on maintenance therapy; 5% stated that they wanted to continue using other illegal substances alongside treatment.

**Table 1:** Demographics for participants in the assessment

Characteristic	Total N (%)	Methadone, N (%)	Buprenorphine, N (%)
Total	122	68	53
Age (years)			
Less than 25 years old	4 (3)	1 (1)	3 (6)
Between 25 and 50 years	98 (80)	57 (84)	40 (75)
More than 50 years	20 (16)	10 (15)	10 (19)
Sex			
Male	89 (73)	48 (71)	41 (77)
Female	33 (27)	20 (29)	12 (23)
Number of years using the substance			
0-5 years	15 (12)	11 (16)	4 (8)
6-10 years	34 (28)	13 (19)	21 (40)
11-15 years	18 (15)	9 (13)	9 (17)
16-20 years	26 (21)	17 (25)	9 (17)
21-25 years	12 (10)	7 (10)	5 (9)
Over 25 years	17 (14)	11 (16)	5 (9)
Use of illegal substances intravenously			
Every day or at least every week	11 (18)	6 (9)	5 (9)
Once/twice a month	8 (7)	3 (4)	5 (9)
Never	103 (84)	59 (87)	43 (81)
Picking up medication			
Everyday	6 (5)	3 (4)	3 (6)
Once a week	43 (35)	26 (38)	17 (32)
Every two weeks	47 (39)	27 (40)	19 (36)
Once a month	17 (14)	6 (9)	11 (21)
Other	9 (7)	6(9)	6 (3)

The majority attend an addiction treatment center for medication collection and/or consumption weekly or monthly (35% weekly, 49% bimonthly, 14% monthly). A small group (5%) beginning treatment for the first time, attend daily. For most patients (93%), the activities associated with medication collection and consumption take less than an hour, 7% report a duration of half the day.

Quality of life and convenience

**Figure 1:**
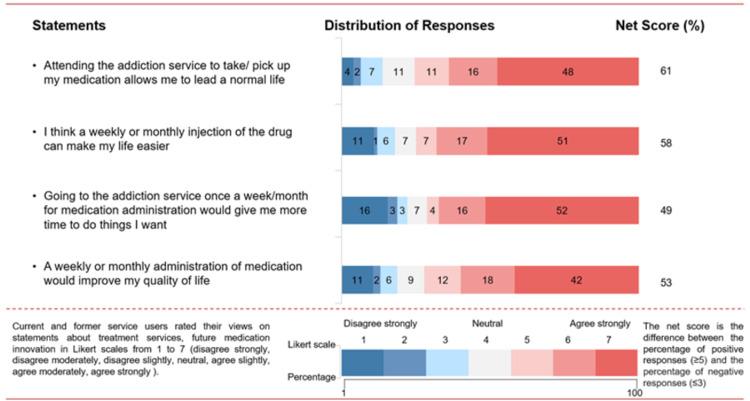
Participants’ views on their quality of life using medication for opioid use disorder

**Figure 2:**
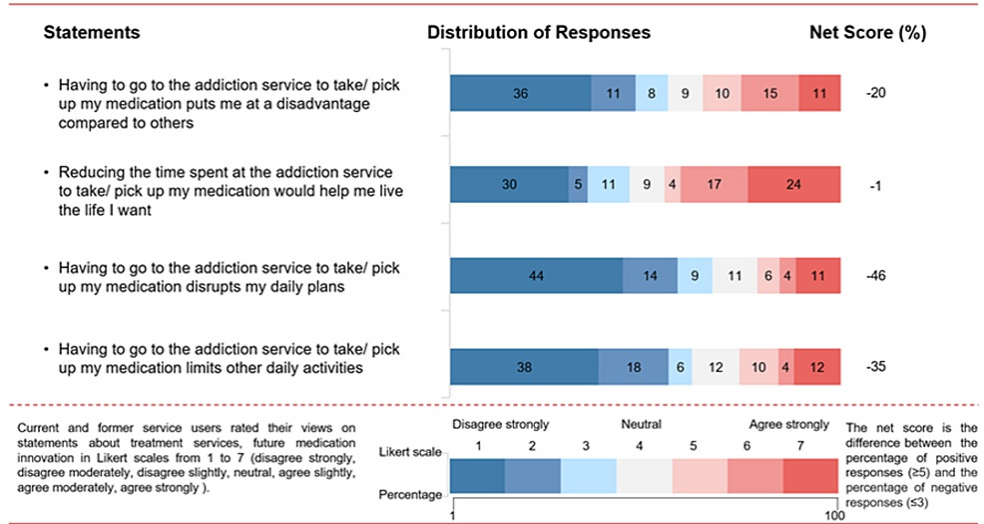
Participants’ views on the convenience of taking medication for opioid use disorder

Seventy-five percent of participants have a positive attitude towards engaging with treatment agreeing that “it allows me to lead a normal life” (net score +61%; Figure 1). Many participants indicated that going to the addiction service was not a major inconvenience (Figure 2). They report medication collection from the addiction service did not disrupt their daily plans (net score −46%) and did not limit them from doing other daily activities (net score −35%). Fifty-five percent of patients believed that having to go to the addiction service to take medication did not disadvantage them compared to others (net score −20%).

The majority (67%) of those who attended the service every day stated it disrupted their daily plans (net score +33%) and daily activities (net score +33%); 83% also agreed that reducing time spent at the addiction service would help them live the life they want (net score +67%).

Most participants stated that current treatment options did not pose significant limitations, but they did believe that novel therapeutic options would improve their treatment experience. Most believed that weekly or monthly administration of treatment injections could make their lives easier (net score +58%). Fifty-one percent of participants considered PRB would have a very significant improvement on their quality of life: weekly or monthly administration of medication would allow for more time to do things they want (net score +49%) and improve their quality of life (net score +53%).

There are differences in calculated scores describing the responses of participants for groups currently treated with methadone or buprenorphine. The assessment of the possible impact of PRB in groups of prescribed methadone indicated that novel therapeutic options would improve their quality of life - making life easier - (net score +72%); the same assessment for that prescribed buprenorphine (net score +39.6%) indicates a difference. This was similar in other assessments such as “Time to do things they want” (net score +58.8% vs +35.8%) and improving their overall quality of life (net score +66.2% vs 35.8%).

Efficacy and clinical

**Figure 3:**
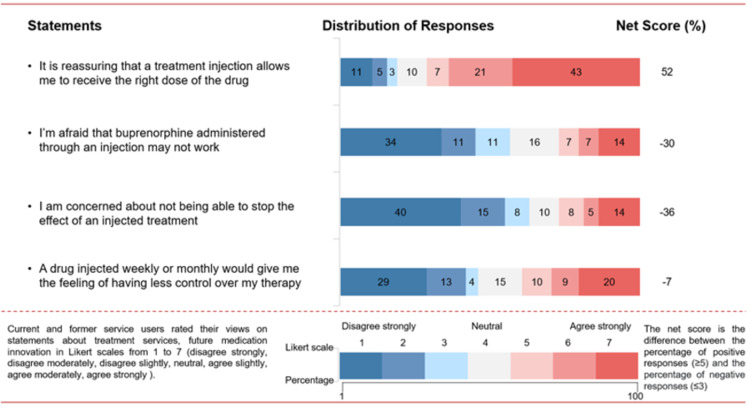
Participants’ views on efficacy and clinical outcomes of buprenorphine

Participants had low levels of concern about limitations in the efficacy of PRB therapy (net score −30%), lack of ability to stop the effect of the treatment (net score −36%), and having less control over their therapy (net score −7%; Figure 3). The majority stated that it is reassuring that the injection treatment allows them to receive the right dose of the drug (net score +52%).

Inequity and stigma

**Figure 4:**
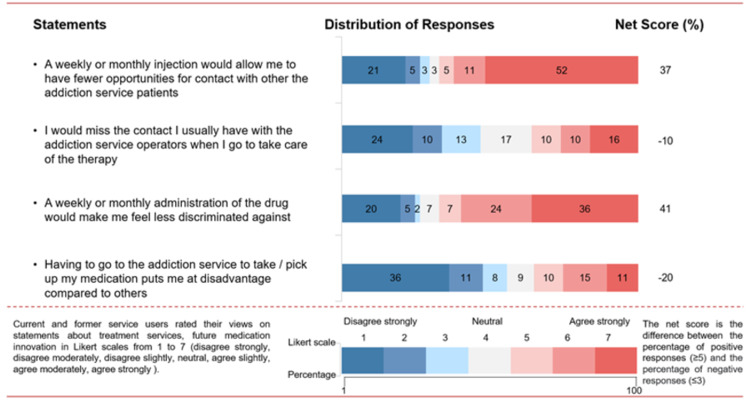
Participants’ views on the inequity and stigma of taking medication for opioid use disorder

The majority (67%) stated that a weekly or monthly administration of the drug would make them feel less discriminated against, avoiding stigma perceived by attending the service (net score +41%; Figure 4). Participants positively report that weekly/monthly attendance would allow them to have fewer opportunities for contact with other addiction service patients (net score +37%) and indicate that contact with addiction service operators would not be missed (net score −10%).

## Discussion

This work is the first and currently largest (1,830 cumulative years of combined treatment experience) lived experience assessment of how the introduction of PRB, a novel therapeutic option, may address current limitations in OUD treatment in Italy.

Results from this assessment describe the likely impact of the introduction of PRB, specifically in addressing the limits of current therapy (reduced quality of life, stigma). Overall, participants have a positive attitude towards engaging with treatment services and indicate that it allows them to lead a normal life. Novel therapeutic options would improve quality of life, reduce stigma and inequity, and allow more time for other activities. Adoption of PRB is likely to reduce the burden of OUD treatment.

The majority were not concerned about efficacy, or control over the new treatment and were not opposed to less frequent contact with treatment services and other patients. It is likely there are few barriers to uptake or concerns surrounding the new PRB.

Response to statements about convenience showed differences between subgroups with different attendance frequencies of treatment services and different current methods of treatment. The subgroup of patients attending the treatment service daily disagreed with the overall distribution of responses, stating that current treatment disrupts their daily life and prevents them from doing other daily activities. The majority of people in this group on frequent medication collection agreed that PRB would improve their quality of life. The group of participants attending services daily to collect medication frequently (5% of the total participants) are at the beginning of their treatment experience, which includes regular contact with services. It is important to understand how such novel therapeutic options are placed for those at the early stages of treatment.

Comparing those participants prescribed methadone medication as their current treatment with those taking buprenorphine, showed a greater expectation for increased quality of life following change to the novel therapeutic option.

Limitations

The data generated were self-reported and susceptible to the social desirability bias in which participants wish to provide answers the experimenter is searching for, or to minimize judgment from others. To prevent the effect of social desirability pressure, the survey was anonymous and conducted privately.

Further studies

This assessment acts as a baseline, and further work should be done on populations who have recently switched over to PRB from conventional oral administration treatment methods. This would reveal the patient-focused experiences about the process of switching treatment strategies as well as changes observed once on the new treatment.

## Conclusions

Current treatment strategies for OUD, although effective, do not fully meet the needs of patients, and their potential benefits are limited due to oral administration methods. The introduction of innovative PRB may reduce negative outcomes and can improve the quality of life and reduce the stigma faced by patients due to less frequent attendance to treatment services. The effect of these benefits would be most felt by those currently attending treatment services on a daily basis, and for those on methadone instead of buprenorphine. Further studies on individuals who have recently switched to novel therapeutic options could provide more insights into a reduction of negative treatment outcomes to guide treatment decisions.
